# Uncovering the cytotoxic effects of air pollution with multi-modal imaging of *in vitro* respiratory models

**DOI:** 10.1098/rsos.221426

**Published:** 2023-04-12

**Authors:** Zeinab Al-Rekabi, Camilla Dondi, Nilofar Faruqui, Nazia S. Siddiqui, Linda Elowsson, Jenny Rissler, Monica Kåredal, Ian Mudway, Anna-Karin Larsson-Callerfelt, Michael Shaw

**Affiliations:** ^1^ Department of Chemical and Biological Sciences, National Physical Laboratory, Teddington, UK; ^2^ Faculty of Medical Sciences, University College London, London, UK; ^3^ Kingston Hospital NHS Foundation Trust, Kingston upon Thames, UK; ^4^ Lung Biology, Department of Experimental Medical Science, Lund University, Lund, Sweden; ^5^ Bioeconomy and Health, RISE Research Institutes of Sweden, Lund, Sweden; ^6^ Ergonomics and Aerosol Technology, Lund University, Lund, Sweden; ^7^ Occupational and Environmental Medicine, Lund University, Lund, Sweden; ^8^ MRC Centre for Environment and Health, Imperial College London, London, UK; ^9^ National Institute of Health Protection Research Unit in Environmental Exposures and Health, London, UK; ^10^ Asthma UK Centre in Allergic Mechanisms of Asthma, London, UK; ^11^ Department of Computer Science, University College London, London, UK

**Keywords:** respiratory toxicology, bioimaging, microscopy, cell and tissue culture models

## Abstract

Annually, an estimated seven million deaths are linked to exposure to airborne pollutants. Despite extensive epidemiological evidence supporting clear associations between poor air quality and a range of short- and long-term health effects, there are considerable gaps in our understanding of the specific mechanisms by which pollutant exposure induces adverse biological responses at the cellular and tissue levels. The development of more complex, predictive, *in vitro* respiratory models, including two- and three-dimensional cell cultures, spheroids, organoids and tissue cultures, along with more realistic aerosol exposure systems, offers new opportunities to investigate the cytotoxic effects of airborne particulates under controlled laboratory conditions. Parallel advances in high-resolution microscopy have resulted in a range of *in vitro* imaging tools capable of visualizing and analysing biological systems across unprecedented scales of length, time and complexity. This article considers state-of-the-art *in vitro* respiratory models and aerosol exposure systems and how they can be interrogated using high-resolution microscopy techniques to investigate cell–pollutant interactions, from the uptake and trafficking of particles to structural and functional modification of subcellular organelles and cells. These data can provide a mechanistic basis from which to advance our understanding of the health effects of airborne particulate pollution and develop improved mitigation measures.

## 1. Introduction

Air pollution is a major global health concern. In 2018, The World Health Organization (WHO) estimated that 90% of the global population lived in areas which fail to meet their guideline concentration for ambient fine particulate matter (PM_2.5_), an annual average of 10 µg m^−3^ [1]. In September 2021, this guideline value was revised down to an annual average of 5 µg m^−3^ reflecting a growing body of the literature demonstrating significant health impacts below 10 µg m^−3^ [2–4]. Moreover, within many indoor environments, exposure to unhealthy levels of particulate pollution resulting from biomass burning, first- and second-hand tobacco smoke and other recreational and occupational irritants often results in concentrations of PM_2.5_ far above outdoor levels, contributing to additional health burdens. From 1990 to 2017, outdoor air pollution was estimated to contribute to 4.6 million premature deaths and a total of 143 million disability-adjusted life years worldwide; predominately reflecting impacts on cardiopulmonary diseases [5]. For comparison, the number of premature deaths associated with air pollution annually exceeds those associated with malaria, HIV infection, cigarette smoking, obesity, alcoholism and drug use [6].

Epidemiological studies demonstrate significant associations between poor air quality and many short- and long-term health impacts, including hospital admissions for various diagnoses, changes in lung growth, impaired lung function, respiratory symptoms, increased prevalence and incidence of asthma, and effects on child birth outcomes and mortality [7]. Acute exposures to particulate and gaseous pollutants exacerbate common respiratory conditions such as asthma and chronic obstructive pulmonary disease (COPD) [8–14]. Longer-term exposures have been associated with reduced lung growth and function [15–17], progression of obstructive airway diseases [18] and premature death [19].

Established evidence suggests that the adverse health effects attributable to particulate pollution depend not only on the physical properties of PM (such as aerodynamic diameter in air, surface area and number) but also its chemical composition. However, it is often challenging to separate the relative contribution of PM constituents from the reported population-level health effects, as many are derived from common sources and are highly correlated in PM. Historically, the levels of atmospheric particulate pollutants have been regulated by the mass concentration of discrete size fractions: PM_10_ and PM_2.5_. However, classifying PM by mass concentration alone ignores the chemical heterogeneity of airborne particles. Further, current regulations do not adequately address the toxicologically important contributions of ultrafine particulate matter (UPM < 100 nm) [20]. As a result, there is a pressing need to understand the contribution of PM constituents to specific toxicological pathways and establish causal links between particles in the air we breathe and downstream health effects. Significant gaps persist in our understanding of the specific mechanisms by which individual pollutants elicit their effects. Exposure to atmospheric air pollution results in a series of acute responses including impaired airway function due to airway sensory afferents [21], initiation of oxidative stress [22], activation of redox-sensitive signalling pathways in pulmonary cells [23], induction of acute neutrophilia and lymphocytosis [24] and modification of innate and adaptive immunity [25]. *In vitro* studies, in which respiratory models are exposed to well-characterized aerosols in the laboratory, are an important source of data to improve our understanding of the cause–effect relationships between exposure to airborne particles and cell/tissue damage. Their value is, however, fundamentally dependent on robust *in vitro* to *in vivo* correlation [26], which requires model biological systems which accurately recapitulate the human respiratory system and methods of delivering controlled aerosol exposure. As ‘the dominant form of analysis of molecules, cells, and tissues across the Life Sciences' [27], imaging techniques are key to our ability to investigate the structural, functional and chemical changes that occur following particulate exposure.

This article considers different *in vitro* respiratory model systems and how they can be interrogated using high-resolution imaging techniques to improve our understanding of respiratory toxicology. After a brief review of cellular responses in the respiratory system to airborne particulate exposure, we discuss the state-of-the-art *in vitro* respiratory models and exposure systems for mimicking *in vivo* aerosol inhalation. We then discuss how these models, and their interaction with particulate pollutants, can be visualized and quantified using different forms of microscopy, including fluorescence techniques such as super-resolution and light sheet microscopies, label-free optical imaging and atomic force and electron microscopies. These methods allow the underlying cytotoxicity pathways and mechanisms to be probed in far greater detail than is possible using conventional high-throughput toxicity screening, offering new possibilities to assess toxic effects at the subcellular and multi-cellular levels, including the uptake and fate of particles and particle-associated chemicals in cells and tissues. We highlight the importance of matching the imaging technique to both the model system and the toxicological pathway of interest and discuss the toxicological insights into the impact of PM exposure which can be afforded by effectively combining these technologies.

## 2. Respiratory responses to aerosol exposure

### 2.1. Inhalation of atmospheric aerosols

Atmospheric aerosols vary significantly in their composition (typically comprising elemental carbon, inorganic salts, organic matter, metals and mineral dust) and size distribution (with particle diameters from a few nanometres to a few micrometres). As a result, it has proven difficult to identify which properties (particle size, number concentration, surface area, black carbon mass concentration, chemical composition) dominate the aetiology and progression of detrimental health effects [20,28–31]. Both PM_2.5_ and UPM can carry toxic compounds, allowing them to pass through the upper respiratory tract (URT) to the lower respiratory tract (LRT), which is lined with ciliated bronchial epithelial cells. Particles then continue to alveolar surfaces, which are lined with a thin layer of alveolar epithelial cells, where they can diffuse through the gas exchange network to the capillaries in the lungs, causing systemic effects and damaging other organs [32].

**Figure 1.**
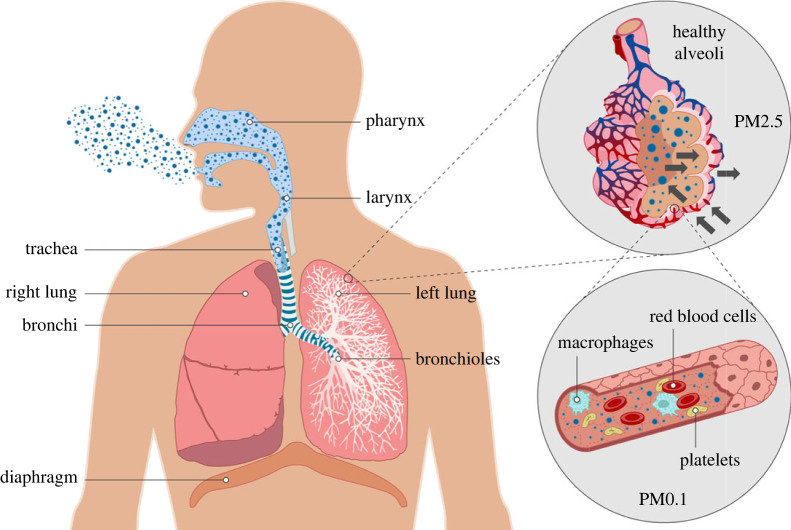
The human respiratory tract and PM inhalation. A mixture of solid particles in the air are inhaled through the URT (nose, nasopharynx and the oropharynx). Typically, the nasal apparatus removes a higher fraction of larger particles (greater than 2.5 µm); however, a certain fraction of these large particles reaches the alveolar section, through ciliary clearance and mucus production from goblet cells. These particles (2.5 µm and less than 1 µm) move down to the LRT (larynx, trachea, bronchi, bronchioles and alveoli), where inhaled air is conducted to the alveolar surface for gas exchange, which takes place between the thin lining of alveolar type I epithelial cells. PMs < 0.1 µm can flow into the blood stream, where they can initiate inflammatory responses and/or affect other organs in the body.

Atmospheric air contains a variety of gases, including sulfur dioxide, nitrogen dioxide, carbon-based gases and ozone [33]. The more water-soluble components (such as SO_2_) are absorbed in the nose and upper airways, with the less water-soluble components (such as O_3_ and NO_2_) reaching the distal airway and gas exchange regions [34] (figure 1). The nasal apparatus typically removes a higher proportion of larger particles (greater than 2.5 µm); however, a fraction of these reach the alveolar regions through ciliary clearance from bronchial epithelial cells and mucus production from goblet cells [34]. The LRT conducts inhaled air to the alveolar surface for gas exchange, which takes place between the thin lining of alveolar type 1 epithelial cells in the alveoli and capillaries. Alveolar macrophages, present at the air–lung interface, remove debris and pathogens that have reached the alveoli [35]. When the LRT is exposed to airborne PM, leucocytes (predominately found in the alveolar capillaries) and epithelial cells secrete cytokines and chemokines that induce inflammation, activate fibroblasts involved in remodelling processes and increase the secretion of signalling molecules such as Ca^2+^ both intra- and extra-cellularly. Abnormally high Ca^2+^ concentrations resulting from exposure to PM_2.5_ have been found to activate a series of inflammatory events, causing elevated free radical production [36] and apoptosis [37].

### 2.2. Oxidative stress

Oxidant air pollutants such as ozone, PM and nitrogen dioxide have been shown to induce lung inflammation through activation of the oxidative stress process [22]. Physiological levels of reactive oxygen species (ROS) are essential for normal cell and organ development and homeostasis, while high levels of ROS induce diverse pathological processes. The presence of oxidant pollutants in the lungs may lead to widespread systemic pathologies, potentially via direct and/or indirect oxidative reactions with molecules and cells present in the airways [38,39], resulting in oxidative stress in airway cells [40]. Previous work has shown that the onset of respiratory conditions like COPD are heavily linked to increased oxidative burden [41].

### 2.3. Inflammatory responses and cell and tissue remodelling

Inflammatory processes play a variety of roles during particle-induced toxicity [42]. Persistent inhalation of toxic airborne particulates stimulates chronic inflammatory responses and, in turn, continuous tissue repair and remodelling. This differs significantly from the injury repair response, where the incessant presence of inflammatory mediators and effector molecules alters the repair process, affecting the composition and remodelling of the extracellular matrix (ECM); ultimately, narrowing the lumen and obstructing the airway [43,44]. Upon interaction with inhaled substances, pulmonary epithelial cells produce inflammatory mediators, especially cytokines, to recruit inflammatory cells. Previous exposure to PM_2.5_ and UPM has been shown to cause an increase in neutrophils in the bronchoalveolar region, suggesting acute inflammation with increased mRNA expression of interleukin IL-6 and IL-10 [45]; whereas repeated/acute exposure to PM_2.5_ and UPM was shown to raise the number of macrophages [45,46] consistent with a shift to a more chronic inflammatory profile. In the subsequent phase, fibrocytes and resident fibroblasts migrate to the inflammatory site and differentiate into myofibroblasts, releasing ECM components causing remodelling of tissue and/or resolving inflammation [47]. Failure of the macrophages to eliminate the threat can lead to the persistent release of mediators causing tissue damage and pro-fibrotic responses. A dysregulated repair process is dictated by cytokines produced by both immune and structural cells. Fibroblasts not only produce ECM components but also secrete inflammatory mediators such as IL-6 and IL-8, regulated on activation, normal T cell expressed and secreted (RANTES), monocyte chemoattractant protein 1 (MCP-1), monokine induced by gamma (MIG) and eotaxin to recruit inflammatory cells [48]. *In vitro* cell studies have shown that exposure to soot, secondary organic aerosols (SOA) and diesel exhaust (DE) particles causes pro-inflammatory responses, including increased release of IL-6 and IL-8 [49]. Bronchial epithelial cells exposed to functionalized carbon black show increased mRNA levels of IL-1β and IL-6 [50] and bronchial epithelial cells exposed to SOA show increased levels of released IL-8 [51]. IL-6 from leucocytes, endothelial cells, keratinocytes and fibroblasts has been shown to play a significant role in acute inflammation and adaptive tissue remodelling [52]. *In vivo* studies in mice have found elevated levels of pro-inflammatory mediators after PM exposure [53,54]. In these studies, elevated levels of MCP-1 were observed which stimulated collagen production in a fibrotic response, favouring matrix deposition in the airways [55]. Mice treated with PM_2.5_ for eight weeks showed an increase in collagen fibre depositions in remodelled vessels [56]. Further analysis of cultured murine vascular smooth muscle cells showed increased intracellular ROS, mediated via nicotinamide adenine dinucleotide phosphate (NADPH) oxidase 1 (NOX1). Mice exposed to PM_2.5_ for five weeks showed a significant increase in the elastic fibre in the lung parenchyma [57]. In addition, an imbalance in proteases primarily released by macrophages caused ECM degradation in the alveolar region resulting in emphysema [43,44]. Inflammatory responses have also been found in epidemiological studies as well as in controlled exposure studies of humans [58]. Exposure to DE has been shown to cause local lung inflammation characterized by neutrophil, lymphocyte and mast cell infiltration, with an upregulation in inflammatory mediators such as IL-8 and adhesion molecules, ICAM-1 and VCAM-1 [59,60]. Furthermore, DE exposure was found to induce systemic inflammation as shown by increased monocyte and leucocyte counts and serum IL-6 [61] and altered proteasome activity of peripheral white blood cells [62] in healthy volunteers exposed to DE under controlled experimental conditions. Likewise, exposure to fine PM has been associated with systemic inflammation characterized by increased levels of cytokines (MCP-1, MIP-1α/β, IL-6 and IL-1β) and adhesion molecules (ICAM-1 and VCAM) observed in blood samples from healthy subjects [63]. Soot exposure has been shown to cause both respiratory as well as cardiovascular health effects [42]. Altogether, IL-6 is acknowledged to be an important biomarker of the acute inflammatory response to particulate pollution. Elevated levels of IL-6 have been observed following PM exposure in *in vitro* [50] and *in vivo* [46,54] studies, as well as in blood samples from healthy volunteers under experimental and real-world exposure conditions [61,63]. This illustrates the relevance and coherence of *in vitro* and *in vivo* toxicology studies to real-world effects in humans.

## 3. *In vitro* models for respiratory toxicology

*In vitro* respiratory models differ significantly in terms of their experimental complexity and capacity to recapitulate structural and functional aspects of the *in vivo* respiratory system. As a result, different models are suited to the investigation of different aspects of respiratory toxicology. Here we consider the key properties of these models (summarized in table 1) and associated systems for laboratory aerosol exposure (summarized in table 2).

**Table 1.** Summary of the principal advantages and disadvantages of different *in vitro* models for respiratory toxicology.

model type	advantages	disadvantages
submerged two-dimensional cell cultures	— easy to prepare monocultures or co-cultures	— fails to recapitulate the three-dimensional *in vivo* environment
	— compatible with fast screening systems	— difficult to mimic real-world exposures
ALI cultures	— mimics the *in vivo* epithelium	— only feasible for studying exposure effects on epithelial cells
	— realistic exposure of cells to a range of aerosols	
	— longer culture periods to study remodelling processes	
	— compatible with advanced co-cultures and a range of cell types	
lung-on-a-chip	— allows study of interactions between airways and vasculature	— isolated system that does not fully incorporate the three-dimensional environment or tissue complexity
	— immune cells can be incorporated in the system	
spheroids and organoids	— recapitulates cell–cell and cell–ECM interactions	— simplified three-dimensional system lacking vascularization and physiological function
	— three-dimensional environment to study epithelial-to-mesenchymal transition	
three-dimensional cell scaffolds	— *in vivo*-like three-dimensional architecture with a mono-, co- or multi-culture of cells	— lacks circulating immune cells
		— limitations in mimicking physiological function
precision cut lung slices	— snapshot of the lung with intact cellular architecture and biological processes	— lacks circulating immune cells
		— shorter lifetime than other models
	— three-dimensional model that most closely resembles the *in vivo* system	— complex and heterogeneous
		— requires fresh lung tissue

**Table 2.** An overview of aerosol exposure systems for *in vitro* toxicology studies.

exposure system	references	deposition mechanism	aerosol generation system
VITROCELL (various systems)	[64,65]	passive (diffusion and sedimentation)	optional
CULTEX (original linear system and updated radial system)	[66–68]	passive (diffusion and sedimentation) or electrostatic deposition (chamber is available in different versions)	optional but generator is available
ALICE (Air–Liquid Interface Cell Exposure system)	[69]	cloud settling/sedimentation	vibrating membrane nebulizer
MicroSprayer	[70,71]	cloud settling/sedimentation	PennCentury
NACIVT (Nano Aerosol Chamber In Vitro Toxicity)	[72,73]	electrostatic deposition	optional
XposeALI (Advanced exposure system)	[74]	passive (diffusion and sedimentation)	may include a generator for dry aerosols
minu cell system	[75,76]	diffusion and convective transport	optional

### 3.1. 2D cell cultures

#### 3.1.1. Submerged 2D cell culture systems

**Figure 2.**
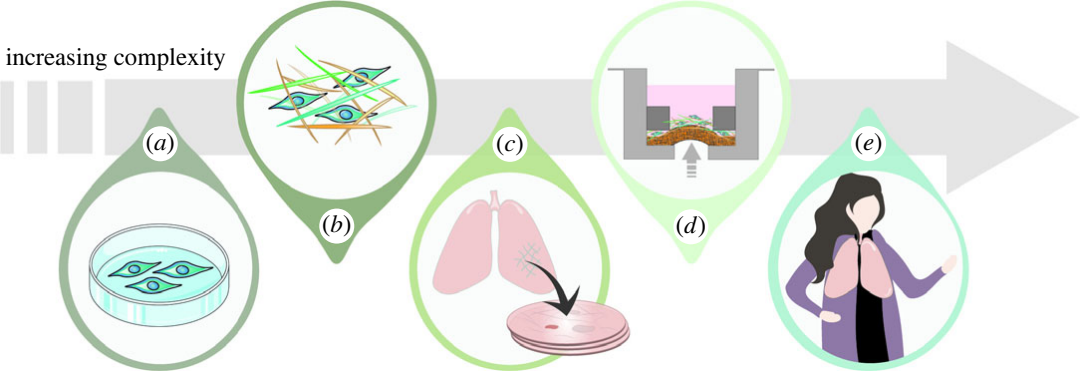
Model systems for respiratory toxicology. (*a*) The commonly employed submerged cell culture system. (*b*) Cells cultured in scaffolds to mimic the native lung composition of ECM. (*c*) Native slice cultures of PCLS with intact lung compartments of airways, vascular and alveolar localizations with structural and inflammatory cells. (*d*) Co-culture systems, PCLS and repopulated lung scaffolds subjected to a dynamic load, which mimics breathing patterns *in vivo*. (*e*) *In vivo* lung parameters in healthy individuals and patients with pulmonary diseases.

For decades, analysis of two-dimensional submerged monocultures in media (figure 2*a*) provided the foundation for understanding the function and diseases of the respiratory tract [77], with particular focus on the airway epithelium. A multitude of primary (human bronchial epithelial cells (HBEC)) and non-primary epithelial cell lines such as A549 (adenocarcinomic human alveolar basal epithelial cells), NCI-H441 (adenocarcinomic distal lung epithelial cells which have barrier properties), Calu-3 (bronchial epithelial cells) and BEAS-2B (non-tumorigenic bronchial epithelial cells) have been derived from the airway epithelium and carcinomas and are widely used [78]. Structural cells such as fibroblasts are traditionally studied in submerged monocultures using primary donor cells, primary human fetal lung fibroblasts (HFL1 or MRC-5 cell lines). These cell lines are derived from fetuses and may offer different responses to adult lung fibroblasts obtained from patients or healthy subjects [79]. Immune responses are commonly assessed using the human leukaemia monocytic cell line THP-1 [80] and the murine monocyte/macrophage-like cell line RAW264.7 [81] to investigate monocyte/macrophage functions in response to particle exposure. In submerged cultures, toxins or compounds such as PM, nanoparticles or drugs, are administered to cells by adding them to the media (see review [79]). These submerged monocultures are practical, reproducible and easily accessible; however, they fail to recapitulate the complex environment of the respiratory tract. Studies of aerosol particle toxicity performed under submerged conditions require particle collection, extraction and dispersion/suspension in relevant media. These processing steps can alter both the physical and chemical properties of the particles [82–84] and can result in the formation of bio-coronas on particle surfaces which differ from those formed as particles transit through lung lining fluid *in vivo* [85,86].

#### 3.1.2. Air–liquid interface cultures

To mimic the air–liquid interface (ALI) of the epithelium, cells can be cultured on top of a porous or permeable membrane that allows the delivery of nutrients from the medium below [87–89]. ALI cultures comprise a pseudostratified columnar epithelium, closely mimicking the *in vivo* epithelium structure [88,90]. Exposures of ALI cultures create a more realistic interface for cell–aerosol interaction, mimicking *in vivo* uptake in the airways [69] wherein aerosolized particles are deposited directly from the air onto the cells. There are several types of aerosol exposure systems available on the market (VITROCELL, CUTLEX, ExposeALI and NACIVT), and several more have been developed by laboratories for their internal use (e.g. ALICE, Minu cell system, MicroSprayer). An overview of some systems frequently used in the scientific literature is given in table 2. A critical factor in developing these systems is the need to maintain a homogeneous and even distribution of particles over the entire cell surface and ensure a reproducible deposition of particles [91]. Another important consideration is the method of direct deposition of the particles to the cell surface. For fine and ultrafine PM, deposition is primarily through diffusion, a mechanism that is not directed specifically to the cell surface. For larger particles, sedimentation is the major deposition mechanism, which can be more easily directed towards the surface; however, this can lead to a relatively small fraction of the particles being deposited onto the cells. In the NACIVT exposure system, a unit for particle charging is combined with a weak electric field over the cell surface to enhance nanoparticle deposition. A version of the CULTEX system has also been developed using electrostatic deposition. For most systems, the deposition efficiencies are generally low for particles entering the exposure chambers, and typically vary considerably with particle size—especially between nanoparticles and larger particles. A low deposition efficiency may lead to uncertain particle doses, a problem that can be solved by measuring the deposited content chemically or by using imaging techniques. However, in the case of generating broad particle size/surface area/mass size distributions, the fact that the deposition mechanisms are size dependent, and that particle agglomeration may occur needs to be accounted for.

### 3.2. 3D cell and tissue culture models

#### 3.2.1. Hydrogels and tissue scaffolds

Hydrogels can be used to create a three-dimensional environment (scaffold) using either biological materials (alginate, lung ECM), synthetic polymers (poly (lactic acid), polyethylene glycol) or a combination of both (figure 2*b*). The use of scaffolds has been shown to augment the phenotypic response of airway epithelial cells [92–94]. Respiratory studies have predominantly used lung ECM-based hydrogels [95]. Decellularized lung tissues, in which cells are chemically removed to leave behind the ECM, provide an alternative means of generating lung scaffolds, with the advantage that they maintain the composition, morphology and mechanical properties of the lung ECM [96,97]. Using cells cultured in decellularized lung scaffolds with specific isotope labelled media (SILAC), it is possible to follow ECM turnover rate and remodelling processes using advanced mass spectrometry to explore cellular events (adhesion, proliferation, differentiation) which affect matrix synthesis and composition [98]. Organotypic models achieve an *in vivo*-like three-dimensional architecture by combining scaffolds with a co- or multi-culture of cells. A combination of different cell types can better replicate the *in vivo* epithelial barrier [99,100] and recapitulate pro-inflammatory and cytotoxic events after exposure [101–103].

#### 3.2.2. Spheroids and organoids

Spheroids are cellular aggregates generated using tumour biopsies or cancer cell lines and are composed of external proliferating, internal quiescent and necrotic zones which recapitulate cell–cell and cell–ECM interactions [104]. Owing to their resemblance to the microenvironment of natural lung tumours, spheroids have been mainly applied in cancer studies and cancer response therapies [105,106], cell-based therapies [107] and pulmonary fibrosis research [108]. Spheroids can be cultured at the air interface [109], indicating potential use for *in vitro* studies to explore the impact of airborne toxins exposure on diseased subjects. Epithelial three-dimensional spheroids have been cultured with BEAS-2B cells to study the effect of DE particles, with results indicating that particles induced epithelial to mesenchymal transition [110].

Organoids are three-dimensional multi-cellular models derived from cultures of stem cells (primary cells, induced pluripotent stem cells and human pluripotent stem cells). As in organogenesis, the constituent cells undergo self-organization into native-like tissue structures, guided by differentiation and specific adhesion properties [111]. They can be cultured in matrix-free or matrix-based systems and have proved a useful tool for studying pulmonary responses [112–114], as disease models [115,116], for developing personalized medicine approaches, in drug screening/discovery [117] and for studying lung development [118]. Although organotypic models represent an advance on conventional monocultures, they still fail to fully represent the complexity of the lung tissue; in particular, they lack vascularization and do not mimic physiological function. Further, there remains a lack of suitable standardized culture methods [119].

#### 3.2.3. Precision cut lung slices

Precision cut lung slices (PCLS) are obtained from healthy or diseased lungs from patients or animal models [120] and have proved a valuable tool for pharmacological and toxicological studies of the distal lung [120–122] (figure 2*c*). The PCLS model represents a snapshot of the *in vivo* condition, in which the three-dimensional organization and cellular composition of airways, vasculature and lung parenchyma are maintained. To generate the slices, lung lobes are filled via the trachea or bronchioles with low-melting agarose and gelatin to fill the vasculature, improving vessel function and visualization. The lung is then chilled to solidify the agarose and gelatin and sectioned into (250–500 µm) slices using a microtome or a vibratome. The slices are then maintained in cell culture media in an incubator. The agarose in larger airways is generally washed away by several changes of the cell medium during the first hours after slicing, whereas some agarose still remains in the parenchyma. The lung slices have preserved functional cilia movements and mucus transport in the airways. Slices are viable for at least 72 h [48,122], and they can be used to study the toxic or inflammatory effects of aerosol exposures [48]. PCLS have many advantages. In total, 30–100 sequential slices can be obtained from a human or animal lung tissue preparation, which dramatically reduces the number of animals needed for experiments. Slices are usually studied in multi-plates with minimal medium solution; they can be viewed under the microscope making it possible to study airways and vessels at different locations from one lung lobe. This results in more specialized screening of exposure responses in different compartments of the lung, helping reduce experimental error and improve correlation with the *in vivo* response. Release of inflammatory mediators from the slices to the media or functional responses involving smooth muscle dilatations or constrictions can be studied simultaneously in airways and vessels by live imaging following pharmacological interventions or aerosol exposures [48,123–125]. To study remodelling processes, PCLS can be exposed to elastase [126] to mimic emphysema and TGf-β [125] or a fibrotic cocktail [127] to mimic fibrosis. The slices can be further analysed by transcriptomics [128] or MS-based proteomics [129]. To study ECM turnover, the slices can be decellularized and remaining versus newly synthesized matrix proteins can be followed and analysed by MS [98]. It is also possible to incorporate stretch to the lung slices mimicking *in vivo* breathing patterns [130–132].

### 3.3. Lung-on-a-chip systems

Lung-on-a-chip systems have been developed using co-cultures of different respiratory cells (epithelial and endothelial cells) cultured in individual microfluidic channels, separated by an elastic permeable membrane that can be cyclically stretched to mimic breathing (figure 2*d*). One side of the membrane contains a fully differentiated epithelium in ALI culture, while on the other, there is an endothelium exposed to a fluid flow. This system has been used for inflammatory studies of chronic lung disorders such as COPD [84] and toxicology analysis [133]. However, despite this sophistication, these systems are limited in their capacity to mimic the three-dimensional environment of the lungs, including structural properties such as stiffness, morphology and ECM composition. There is increasing evidence that the microenvironment has a more significant role in cell fate than previously thought [96,134]. The altered structural properties due to either build-up of fibrotic stiff tissue or the loss of structural support (emphysema) have an impact on cell–ECM and cell–cell signalling, highlighting the importance of carrying out mechanistic studies in relevant cell culture models.

## 4. High-resolution imaging of respiratory models

**Figure 3.**
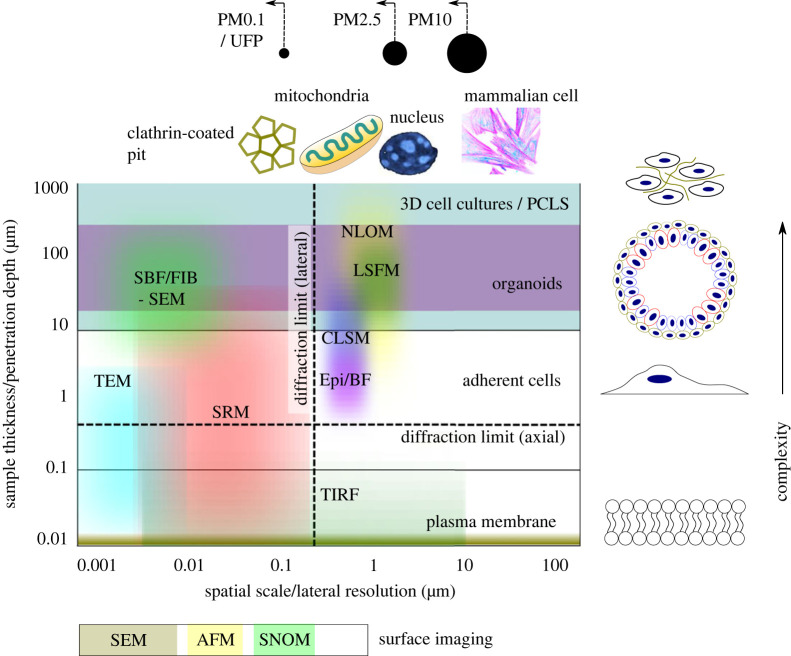
Practical and theoretical limits to *in vitro* imaging. Schematic diagram illustrating the spatial resolution and depth penetration of different *in vitro* imaging techniques and how these relate to the size of relevant subcellular and cellular structures, PM size ranges (top) and respiratory model systems (right).

Imaging techniques can be applied to visualize and quantify the uptake of PM providing insights into actual cellular dose, the trafficking pathways [135] and how exposure perturbs structural and functional properties and the distribution of biomolecules within respiratory models. We consider different classes of high-resolution microscopy techniques in terms of their capacity to probe aspects of cell–PM interactions and induced cytotoxicity in different respiratory models: light microscopy (LM), including fluorescence and Raman techniques; electron microscopy (EM) and scanning probe microscopy (SPM). Figure 3 illustrates the spatial resolution and sample depth penetration achievable with some of these techniques and how they relate to the size of PM, cells and organelles and the dimensions of model systems.

### 4.1. General considerations for *in vitro* microscopic imaging of respiratory models

#### 4.1.1. Spatial resolution, sample size and image contrast

Increasing the spatial resolution of biological imaging techniques has been the focus of significant efforts in recent years, which has led to new methods and improved hardware, probes, sample preparation and image processing methods. Super-resolution fluorescence (SRM) [136] and cryo-electron microscopy [137] in particular allow visualization of the distribution of biomolecules, and cellular and particle ultrastructure at scales previously impossible under conditions closer to their native state. In aerosol–cell interactions, the required spatial resolution is determined both by the size of the PM and the cellular components of relevance. Common features of interest for respiratory toxicology include the cellular machinery involved in endocytosis such as endocytic membrane protein including clathrin-coated plaques and pits (approx. 100–200 nm) [138] and cavolae (approx. 50 nm) [139], endocytic vesicles (approx. 50–150 nm) such as clathrin-coated vesicles [139] and endosomes (approx. 100–500 nm) [140], as well as larger organelles such as mitochondria (approx. 0.5–3 µm), the cell nucleus (approx. 6 µm) and entire eukaryotic cells (approx. 10–100 µm).

The size and relatively high electron density of many aerosol particles such as soot, metals and silica mean they are often directly visible in EM without the need for additional labelling [135]. Exogenous staining methods such as metallic sputter coating, negative staining [141] and immunolabelling [142] are widely used to increase the contrast and specificity in EM imaging of native biological structures. In some cases, particles can be visualized directly using bright-field or phase contrast-based optical microscopy [143]. However, smaller particles, such as those with diameters significantly below the diffraction limit, and which weakly scatter or absorb light can be difficult to detect without exogenous labelling. Fluorescence microscopy (FM) is widely used to visualize PM [144] alongside fluorescently tagged biomolecules of interest. Fluorescent reporter dyes are also employed to detect toxicologically relevant readouts such as ROS [145], intracellular Ca^2+^ ion concentration [146] and cell viability [147].

#### 4.1.2. Depth penetration

Imaging at depth inside a three-dimensional system requires some form of axial sectioning to remove out-of-focus details. This can be achieved virtually, as in a confocal microscope, or physically, via the preparation of thin sample sections, as in resin embedded- or cryo-sections prepared using a microtome for transmission electron microscopy (TEM). Signal attenuation caused by scattering and absorption are particularly problematic in three-dimensional optical imaging of respiratory model systems such as cell-seeded scaffolds, organoids and spheroids. The use of longer wavelengths and tissue clearing [148], in which the sample is processed to remove constituents such as lipids and immersed in a buffer which reduces the magnitude of refractive index differences, can significantly improve depth penetration. Used in combination, these approaches can enable high-resolution imaging throughout thick, highly scattering samples such as intact murine lungs [149].

#### 4.1.3. Live cell imaging: temporal resolution and phototoxicity

Visualizing rapid dynamic events such as the motion of aerosol particles diffusing through a submerged model system requires relatively high imaging frame rates. At physiological temperature, the mean squared displacement of a 100 nm diameter spherical particle moves approximately 3 µm in 1 s. Active transport of internalized particles along microtubules occurs at comparable velocities of approximately 4 µm s^−1^ [150]. To avoid any perceptible motion blur, the exposure time should be less than the smallest resolvable distance divided by particle velocity; for an optical microscope with a 1.3 numerical aperture (NA) objective tracking green fluorescent particles (emission maximum approx. 520 nm, Abbé cut-off frequency of 200 nm) this means a maximum exposure time of 50 ms. Exposing living cells to light can result in phototoxic responses, including the formation of reactive photochemical species, increased temperature and DNA damage. Phototoxicity is exacerbated by the use of fluorescent probes, the excitation of which results in the formation of free radicals such as ROS which react with endogenous biomolecules [151]. Understanding these effects, and conducting appropriate controls, is particularly important when using *in vitro* imaging to study the effects of aerosols and disentangle the effects of phototoxicity from the cytotoxic effects of PM exposure.

### 4.2. Imaging methods for 2D respiratory models: basal membranes of cultured cells and ultrathin tissue sections

#### 4.2.1. Transmission electron microscopy

**Figure 4.**
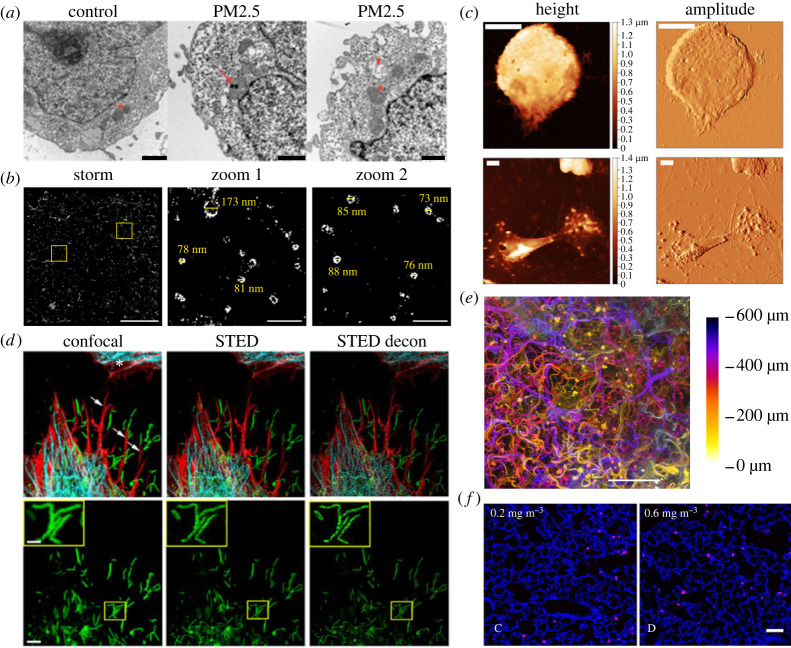
Example of high-resolution images of respiratory models. (*a*) Internalization of PM_2.5_ within the inner mitochondrial membrane in HTR-8 cells (arrow) and mitochondrial vacuolization revealed by TEM. Scale bars are 1 µm. Adapted from Naav *et al.* ([154], fig. 6A–C); copyright 2020; https://creativecommons.org/licenses/by/4.0/. (*b*) Ring-shaped structures of CD81 in the membrane of IgG-activated lung macrophages revealed using STORM. Scale bars are 5 μm in main image and 0.5 μm in zoomed images. Adapted from Ambrose *et al.* ([155], fig. 7B); copyright 2020, Elsevier Group; https://creativecommons.org/licenses/by/4.0/. (*c*) AFM topography images reveal morphological changes in activated eosinophils in individuals with acute asthma (bottom) compared with those from a health control (top). Scale bars are 5 µm. Adapted from Eaton *et al.* ([156], fig. 1*d,e*,*g,h*); copyright 2019, Frontiers in Physiology; https://creativecommons.org/licenses/by/4.0/. (*d*) Visualization of RSV particles (green) in cultured A549 cells stained for F-actin (red) and tubulin (cyan) using confocal microscopy (left), STED (middle) and deconvolved STED microscopy (right). Bottom row shows zoomed in view of part of the cell shown in the top row (RSV channel only). Scale bars are 5 µm in main image and 1 µm in inset image. Adapted from Mehedi *et al.* ([157], fig. 1 Merge and RSV F); copyright 2017, Bio-Protocol via PMC. (*e*) Colour-coded depth projection of a human-derived lung tissue scaffold. Image computed from a focal plane image series captured using an Airy beam light sheet fluorescence microscope. Scale bar is 100 µm; previously unpublished. (*f*) Multi-wall carbon nanotubes (pink) visible in a murine lung section (blue) imaged using stimulated Raman scattering. Scale bar is 50 µm. Adapted from Migliaccio *et al.* ([158], fig. 1C,D; Copyright 2021, Springer Nature Group; https://creativecommons.org/licenses/by/4.0/.

TEM allows ultra high-resolution structural imaging of fixed cells and tissues. Post fixation, samples are embedded in a solid resin which is then sliced into thin (typically less than 100 nm) sections. To improve image contrast, heavy metal stains such as uranyl acetate and lead citrate are used to increase variations in electron density. Electron tomography (ET), in which a series of TEM images are captured as the sample is tilted relative to the incident electron beam, can provide three-dimensional ultrastructural detail of sections (approx. 200 nm to approx. 1 µm thick) with nm level axial resolution [152]. EM techniques are used extensively for structural imaging of the lung and to visualize cellular particle uptake and localization in different *in vitro* and *ex vivo* lung models [125,153]. TEM has been applied to visualize both the effects of PM exposure and to localize intracellular PM. One study [154] used TEM to visualize intracellular uptake of particles in a culture of human trophoblast cells (HTR-8) exposed to a sample of urban PM_2.5_ (figure 4*a*). In addition to gross changes to the appearance of the cell-indicating necrosis, the results showed that PM_2.5_ localize to the mitochondria after 24 h of exposure and result in significant mitochondrial vacuolization and membrane disruption. Cryo-EM [137], in which samples are rapidly frozen and maintained at cryogenic temperatures, offers the potential for imaging cells and biomolecules closer to their native state, free from fixation artefacts. Cryo-EM and cryo-ET are also used for two- and three-dimensional structural and morphological characterization of ultrafine nanoparticles [159].

#### 4.2.2. Fluorescence microscopy: total internal reflectance fluorescence, highly inclined and laminated optical sheet and single-molecule localization

Although capable of spatial resolution which is typically 1–2 orders of magnitude lower than EM, LM techniques offer significant advantages for toxicological imaging due to their compatibility with live samples and the availability of a wide range of fluorescent tags and reporters. For imaging thin samples, total internal reflectance fluorescence (TIRF) microscopy [160] offers a way to significantly improve axial resolution and increase image contrast, using supercritical angle illumination to generate evanescent illumination to limit fluorescence excitation to a short distance (typically 50–300 nm) beyond the sample substrate. TIRF methods are particularly useful for studying dynamic events occurring in the vicinity of the basal membranes of adherent cells. TIRF has been applied to study exocytosis of ATP-loaded vesicles in A549 cells and investigate how the process is affected by hypotonic stress [161]. Highly inclined and laminated optical sheet (HILO) microscopy [162] is a complementary approach which allows imaging deeper into a sample (such as a single cell) by reducing the illumination angle below the critical angle to create a freely propagating, highly angled excitation beam.

TIRF and HILO methods are often combined with single-molecule imaging methods for particle tracking and super-resolved single-molecule localization microscopy (SMLM). SMLM is a class of super-resolution FM techniques based on switching most of the fluorescent dye molecules in a sample into a non-fluorescent dark (off) state such that a small number of spatially distinct molecules are visible in each image frame. The positions of these individual molecules can be localized to within approximately 20 nm laterally, with a composite molecular map or super-resolved image formed by combining data from an image sequence comprising several thousand image frames within which different subsets of molecules are in the ‘on’ state. This ‘on/off’ switching can be achieved in a variety of ways [163] with popular approaches including (direct) stochastic optical reconstruction microscopy (dSTORM or STORM), photoactivated localization microscopy (PALM) and point accumulation for imaging by nanoscale topography (PAINT). The principal downside of SMLM methods is the relatively slow imaging speed and a requirement for relatively sparse fluorescent labelling. In practice, SMLM methods are typically limited to imaging proteins *in vitro* or cells grown on glass substrates. A related class of methods, super-resolution optical fluctuation imaging (SOFI) [164] and super-resolution radial fluctuations rely on post-processing of time-lapse image series to increase spatial resolution by exploiting temporal fluctuations in emitted fluorescence. These methods can accommodate samples with significantly higher fluorophore labelling density; however, they offer a more modest gain in spatial resolution. STORM has been applied to visualize changes in the nanoscale membrane topology of lung macrophages and the spatial distributions of major histocompatibility complex (MHC) class I proteins, [155] (figure 4*b*). Image data showed the formation of membrane projections tipped with these proteins following activation of the cells via Fc receptors by addition of human immunoglobin G antibody. Importantly, the SMLM image data allowed quantification of the nanoscale organization of MHC class I proteins in the membrane and revealed the formation of (sub-diffraction) ring-shaped structures, visualized by fluorescent tagging of the extracellular vesicle marker CD81, which formed upon macrophage activation. Other studies have employed STORM to investigate differences in bacteria phagocytosis between lung macrophages from non-smokers, ex-smokers and COPD patients [165], finding that significantly more bacteria could be detected in SMLM images compared with wide-field microscopy enabling the study to be performed with orders of magnitude fewer cells than would be required using a fluorescence plate reader or fluorescently activated cell sorter.

### 4.3. Surface imaging techniques

#### 4.3.1. Atomic force microscopy

AFM relies on detecting the forces acting between a sharp probe (tip), attached to a flexible cantilever and a sample. Laser light reflected from the back of a cantilever onto a photodiode array allows precise measurement of the deflection of the cantilever as it comes into close contact with a surface [166]. The known stiffness of the cantilever enables this deflection to be related to the interaction force and, by modelling the measured force-indentation response, the reduced elastic modulus and adhesion forces (nanomechanical properties) of a sample can be measured [167]. AFM nanoindentation studies have been conducted on idiopathic pulmonary fibrosis and normal lung tissue biopsies [168], with results indicating that fibrotic lung tissue appeared significantly stiffer than normal healthy lung tissue [168]. *In situ* functional assessment of a variety of human muscle fibres [169] found that AFM measurements could reliably distinguish biopsies from both Duchene muscular dystrophy and Becker muscular dystrophy patients when compared with matched healthy individuals [169]. AFM has been employed to investigate morphological features of cells [170], where it can be used to evaluate morphological differences between asthmatic and healthy individuals [156]. In symptomatic asthmatic whole blood samples, it was shown that many highly activated eosinophils were present, with AFM identifying irregular spreading with multiple pseudopods when compared with the healthy samples, which appeared more round with less spreading [156] (figure 4*c*). Eosinophil recruitment and the presence of granular proteins are considered biomarkers for monitoring allergic inflammatory disorders, such as asthma [171]. Another study identified differences in elastic modulus measurements of bronchial tissue biopsies samples collected from patients suffering from asthma [172]. Combined AFM and FM have been employed to compare the nanomechanical properties and F-actin cytoskeleton of human bronchial fibroblasts from asthmatic and non-asthmatic donors [173]. AFM has also been employed to investigate the morphological and nanomechanical properties of PM_2.5_ [174]. By linking the observable features to mechanical parameters, multi-frequency AFM can be employed to map the viscoelastic properties of biological samples [175–179]. This can be used to investigate variations in viscoelasticity of asthmatic smooth muscle cells with and without the application of pharmaceutical agents. High-speed AFM allows the acquisition of images and spatial resolved biomechanics at rates above 100 Hz (greater than or equal to 1 frame per second, 100 × 100 pixels) [180].

#### 4.3.2. Other scanning probe techniques

In addition to AFM, there are a variety of other scanning probe techniques for characterization of biological interfaces which can be applied to high-resolution topographic imaging of nanoparticles, two-dimensional cell cultures and thin tissue sections. In scanning near-field optical microscopy (SNOM or NSOM), a tip is brought into close contact (a distance significantly less than the wavelength of the incident light) with the sample surface to collect light scattered into the near field [181]. This allows imaging using different contrast mechanisms, including infrared absorption and scattering [182], fluorescence and Raman spectroscopy, with a lateral spatial resolution significantly below the classical diffraction limit. SNOM has been applied to visualize morphological changes in live unlabelled endothelial cells following stimulation with the inflammatory cytokine TNF-α [183]. A combination of AFM, confocal laser scanning microscopy and SNOM have been used to study the distribution of liquid phases in a model of the lung surfactant layer which lines alveolar pathways and is crucial for healthy respiration [184]. These methods enabled visualization of the spatial distribution of fluorescently tagged peptides corresponding to the terminus of surfactant protein B (SP-B), showing how the peptide influences the spatial organization of the two phases.

Tip-enhanced Raman spectroscopy (TERS) is a closely related technique which combines the high spatial resolution of SNOM and AFM with the chemical specificity of Raman spectroscopy [185]. A sharp metal SPM tip in close proximity to the sample surface creates a local enhancement in the Raman signal allowing chemical imaging with molecular specificity with a spatial resolution beyond the diffraction limit. TERS can be used to explore ligand binding to cell membrane receptors [186]. Scanning ion conductance microscopy (SICM) [187] uses a nanopipette filled with electrolyte, with the distance between the pipette tip and the sample surface inferred from the ion current generated between a reference electrode in the sample buffer and a second electrode inside the pipette. SICM has been applied for visualization of interactions between nanoparticles and live human alveolar epithelial cells [188]. In this case, combining SICM with FM enabled exploration of the dynamic interaction between carboxyl-modified latex particles and clathrin-coated membrane structures.

#### 4.3.3. Scanning electron microscopy

SEM images are built up by collecting backscattered electrons (BSE) or secondary electrons (SE) emitted from a sample as a low energy beam of electrons is scanned across it. A lateral spatial resolution of a few nanometres is routinely achievable, and SEM is widely used to characterize the size and shape of a range of nanoparticles [189] including those found in ambient aerosols [190]. Environmental or atmospheric SEM allows imaging of hydrated samples such as cells [191]; however, the generation of reactive species following exposure to the electron beam makes live imaging challenging [192]. As with TEM, cryogenic freezing allows preparation of samples for SEM in a form which is closer to their native state. SEM has been used extensively to explore the ultrastructure of the lung [193]. SEM can be used for visualization of nanoparticles adsorbed onto cell membranes [194] and, although primarily a method for visualizing surface topography, the difference in depth penetration between BSE and SE imaging modes can provide information about the intracellular internalization of nanoparticles by cultured cells [195]. Field emission SEM has been applied to visualize and analyse the adsorption and uptake of metal oxide nanoparticles into cells [196]. When coupled with spectroscopic techniques such as energy dispersive X-ray spectroscopy (EDX) SEM allows spatial analysis of the elemental and chemical composition of PM [197]. Such techniques have been used to characterize ambient PM [198] and to explore links between exposure to PM and a variety of health conditions [199].

### 4.4. Imaging methods for 2.5-dimensional models: cultured cell monolayers, tissue sections

#### 4.4.1. Wide-field and structured illumination fluorescence microscopy

Wide-field systems represent the simplest of FM in which an image of the sample (uniformly illuminated across the focal plane of the objective lens) is captured using a digital camera. Imaging speed is limited by the frame rate of the camera and can be more than several hundred frames per second using a modern scientific camera, such as those with scientific complementary metal oxide (sCMOS) sensors. Wide-field systems are well suited for fast imaging of relatively thin samples such as cell monolayers. However, they are not generally compatible with thicker samples, owing to their fundamental inability to capture axial information [200]. The problem can be overcome to a limited degree using three-dimensional deconvolution [201], in which out-of-focus information in a focal series (*z*-stack) of wide-field images is computationally reassigned using knowledge of the point spread function of the microscope. However, photon (shot) noise typically limits the effectiveness of this approach to relatively thin samples. Structured illumination microscopy (SIM) is a variant of wide-field FM in which the sample is illuminated by spatially modulated excitation light [157]. The effect of this is to shift normally unobservable, high spatial frequency information into the passband of the microscope, allowing recovery of a super-resolved image and removal of out-of-focus light. Typically, this allows a doubling of lateral [202] and axial [200] spatial resolution, reaching close to 100 nm laterally and better than 300 nm axially. Importantly the series of raw images (nine for two-dimensional SIM or 15 for three-dimensional SIM) required in SIM can be captured fast enough for live imaging of dynamic systems [203]. The combination of high spatio-temporal resolution and compatibility with conventional sample preparation and labelling make SIM well suited to visualization of intracellular uptake of nanoparticles as well as co-localization studies to explore particle uptake pathways [204]. It is possible to further increase spatial resolution by inducing a nonlinear response to fluorescence excitation using photo-switchable fluorescent dyes or by saturating the excited state of the fluorescent molecule [205]. To date, there have been relatively few practical biological applications of nonlinear SIM, owing in part to the potential for image artefacts associated with insufficient signal-to-noise for the higher-order passbands. Although often used for imaging of cultured cells on planar substrates and thin tissues, SIM can also be used for super-resolution imaging of thicker, sparsely fluorescent samples [203].

#### 4.4.2. Bright-field and phase microscopy

Despite its lack of specificity, bright-field microscopy remains a ubiquitous and useful tool for minimally invasive biological imaging. For weakly absorbing phase objects such as adherent cells, phase imaging techniques, such as phase and differential interference contrast, provide superior image contrast, however, are typically not quantitative. Quantitative phase imaging (QPI) microscopy [206] provides a map of the optical path length delay introduced as light travels through the sample. In addition to allowing high contrast imaging of cellular and subcellular structures, QPI methods can also be used to measure cell volume and mass and infer functional characteristics such as neuronal activity and intracellular ion concentrations [206]. Traditionally, QPI techniques are limited to imaging thin, weakly scattered samples, such as adherent cells. For example, QPI has been applied for label-free visualization of A549 cells infected with the influenza virus, with individual viral particles visible [207]. More recently, QPI schemes have been proposed for three-dimensional imaging of thicker samples [208]; however, the assumption of weak scattering limits the range of samples for which these approaches are suitable.

### 4.5. Methods for three-dimensional cell cultures, spheroids and organoids

#### 4.5.1. Confocal, light sheet and super-resolution fluorescence microscopy

Confocal laser scanning microscopy (CLSM) [209] is the most widely used FM technique for imaging of thicker samples such as three-dimensional cell cultures. CLSM uses a pinhole to physically block light originating away from the focal plane and create an optically sectioned image. Each two-dimensional image plane is built up sequentially by scanning a focused spot through the sample and three-dimensional images are built up by axially displacing the objective lens or sample to create a focal series. This highly sequential image capture limits the frame rate and the suitability of CLSM for visualizing fast dynamic events, such as particle diffusion, in three dimensions. High-resolution three-dimensional imaging of large samples can often take several minutes or even hours. A spinning disc confocal microscope (SDCM) multiplexes this approach, typically using an array of micro-lenses, to create multiple focal spots which are rapidly scanned over the sample and imaged back through a complementary array of pinholes [210]. At the expense of flexibility and spatial resolution, this can increase image acquisition rate by several orders of magnitude, making the technique well suited for live cell imaging. SDCM has been used to track the internalization of nanoparticles in living cells over time [211]. CLSM has been used to visualize the deposition of inhaled PM_2.5_ in lungs and other organs by imaging tissue sections from mice exposed to aerosolized fluorescent polystyrene particles via a ventilator [212]. Image data showed highly non-uniform PM deposition in the lungs, with particles also found in the kidney and liver. CLSM is also able to provide high-resolution structural images of *in vitro* lung models such as organoids [213] and PCLS [214]. Using the highest numerical aperture objective lenses, a confocal microscope can achieve an axial resolution of approximately 0.5 µm and as such can provide three-dimensional image information even for thinner (2.5-dimensional) samples such as adherent cell monolayers [215]. However, for such samples, alternative FM techniques, including wide-field deconvolution and SIM, can offer significant advantages in terms of imaging speed, light dose and spatial resolution.

Stimulated emission depletion (STED) [216] and related techniques are point-scanning methods similar to CLSM which rely on the use of a doughnut-shaped depletion beam, superimposed onto the diffraction-limited excitation focus, to de-excite fluorophores leaving a small (sub-diffraction) emitting area (or volume) within the sample. By tuning the power of the depletion beam, spatial resolution of 50 nm or even less is achievable. As with CLSM, STED is suitable for three-dimensional imaging relatively deep into thick samples; however, it requires careful selection of fluorescent dyes, and the fluorophore must have significant spectral emission at the wavelength of the depletion beam. STED also requires a relatively high photon flux which can result in sample damage. STED has been used to visualize human respiratory syncytial viral (RSV) particles in A549 cells [157] (figure 4*d*), where the co-localization of RSV particles with filopodia rich in F-actin suggests that filopodia have a role in virus particle transmission between cells.

Light sheet microscopy (LSM) or selective plane illumination microscopy (SPIM) overcomes some of the limitations inherent in other fluorescence imaging techniques including degradation of images due to out-of-focus light, excessive sample light exposure [217] and long image acquisition times by restricting excitation to a thin sheet or plane [218]. Typically, LSM systems make use of a pair of objective lenses mounted perpendicularly; one to project a thin sheet of illumination into the sample and the other to collect emitted fluorescence [219]. A fast scientific camera records a series of two-dimensional images as the sample, or sheet and camera, are scanned perpendicular to the plane of illumination. Traditionally, LSMs use water dipping objective lenses to image a sample immobilized in a hydrogel such as agarose [219]. While well suited for some imaging applications, notably in developmental biology [220], this mounting geometry is often not ideal for toxicological screening applications. Recent LSM systems [221–223] have been developed to make use of an inverted geometry compatible with a wider range of sample-mounted formats including multi-well plates. SPIM and LSM remain a particularly active area of development in FM, with innovations such as excitation beam shaping able to improve the spatial resolution [224] for time-lapse subcellular imaging and the field of view [225] for large-scale volumetric imaging. LSM methods are particularly well suited for imaging deeper into larger, thicker samples such as three-dimensional cell cultures, organoids, model organisms and *ex vivo* tissues. Owing to faster imaging speeds and greater depth penetration, LSM makes it feasible to perform fluorescence imaging of entire organs such as the entire lobe of a mouse lung, which has been imaged in various pathological states [226]. LSM has been used to visualize and localize fluorescent nanoparticles introduced into murine lungs via intratracheal instillation or direct inhalation [227]. LSM is also particularly well suited to volumetric imaging of lung tissue scaffolds, where the autofluorescence of the scaffold allows direct visualization of the scaffold fibres (figure 4*e*).

#### 4.5.2. Nonlinear optical imaging: multi-photon fluorescence, Raman and harmonic generation microscopy

Nonlinear optical microscopy (NLOM) encompasses a class of label-free and label-reliant imaging techniques based on nonlinear interactions between light and a material to generate chemically specific image contrast. NLOM techniques rely on excitation of the sample using a short-pulsed near-infrared laser source which is less prone to scattering in biological samples than the visible wavelengths used in conventional optical microscopy, and as a result can significantly improve imaging depth penetration. The nonlinear dependence of the emission intensity on the excitation irradiance also gives these methods an inherent optical sectioning property making them well suited to three-dimensional imaging.

Multi-photon fluorescence microscopy (MPFM) [228] combines the advantages of FM (molecular specificity, high image contrast) with significantly increased depth penetration, with imaging depths of approximately up to 500 µm achievable in many studies [229]. However, the need for high peak excitation powers also means that laser-induced phototoxicity and sample heating can be significant problems, limiting the application of MFM for time-lapse imaging of live samples. Two-photon fluorescence microscopy (2PFM) is widely used for *in vivo* fluorescence imaging, and it has been used extensively for intravital imaging of the lung [230]. For example, 2PFM microscopy has been used to study PM_2.5_ deposition rates in mice exposed to fluorescence particles via a ventilator [212]. However, the method is also well suited to imaging thick *in vitro* models. In common with other NLOM systems 2PFM systems tend to be point scanning, which limits their image acquisition rate. An exception to this are systems which exploit temporal focusing [231], in which illumination is spectrally dispersed at the pupil of the objective lens resulting in an axial confinement of the emitted fluorescence, which allows fast wide-field 2PFM microscopy. More recently, three-photon excitation (3PFM) has shown promise for *in vivo* imaging with even greater depth penetration using longer wavelength excitation sources [232].

Raman microscopy techniques are based on the generation of sample contrast by inelastic scattering of light [233]. As the resulting wavelength shift depends on the vibrational energy states of the molecules in the sample, analysing the Raman spectrum allows identification of specific chemical bonds. This capacity for label-free chemical imaging makes Raman microscopy attractive for numerous applications. The spontaneous Raman signal is typically very weak, making it generally impractical for imaging; however, two Raman-based methods coherent anti-Stokes Raman spectroscopy (CARS) and stimulated Raman spectroscopy are now widely used for microscopic imaging. Both methods rely on enhancing the Raman signal by tuning the frequency difference between two illumination beams (pump and Stokes) to match a specific vibrational transition in the sample [234]. *Ex vivo* imaging studies of murine tissue sections have shown the potential for label-free visualization of inhaled PM, such as multi-walled carbon nanotubes [158] (figure 4*f*), in different organs. This capacity to reveal the spatial localization of aerosol particles without the need for exogenous labelling makes Raman methods particularly interesting for *in vitro* respiratory toxicology studies.

Harmonic generation microscopy [235,236] relies on the frequency conversion which occurs when a material with a nonlinear susceptibility (polarization response) is illuminated at high optical intensity. Second harmonic generation (SHG), in which two lower energy photons are upconverted to a single high-energy photon with twice the energy, occurs in ordered non-centrosymmetric materials. Notably fibrillar collagen, a key component of the ECM, exhibits a strong SHG response [237] which can be exploited for label-free visualization tissue remodelling in response to PM exposure and structural imaging of lung tissue scaffolds [238] and other collagen-based hydrogels [239]. SHG microscopy has also been applied to visualize other biomolecular assemblies such as microtubules [240]. Third harmonic generation (THG), in which three photons are upconverted to a single photon with three times the energy, occurs where there is a change in refractive index, such as water–lipid and water–protein interfaces. THG microscopy can be used to visualize tissue and cell architecture [241] and, as with SHG, the energy-conserving nature of the signal generation makes it particularly well suited to live imaging [242].

#### 4.5.3. Volume electron microscopy

Both SEM and TEM are inherently limited in their capacity to image at depth. Volume EM methods use serial imaging to allow three-dimensional high-resolution imaging of thicker samples [152] and have been used extensively for analysing the ultrastructure of the lung [243]. Historically, TEM analysis of thin serial sections has been used to reconstruct the three-dimensional structure of thick biological samples, including notably the nervous system of the model organism *Caenorhabditis elegans* [244]. In serial block face (SBF) and focused ion beam (FIB) SEM, the specimen surface is repeatedly imaged as thin sections are removed using either a diamond knife or a focused (gallium) ion beam. Lateral resolution down to a few nanometres and an axial resolution (slice thickness) from a few nanometres to over 100 nm depending on the sample and method. SBF and FIB-SEM can be used for structural imaging of large thick samples such as tissues [245], three-dimensional scaffolds [246] and spheroids [247], with one recent study applying FIB-SEM to visualize the uptake of ultrafine magnetic nanoparticles in a three-dimensional spheroid culture model [248].

### 4.6. Emerging methods

Several recent developments in optical microscopy show promise for *in vitro* visualization of the intracellular uptake of respiratory toxins and the subsequent biological response. Computational microscopy [249] techniques combine optical sensing with algorithmic image reconstruction to improve aspects of microscope performance. Such approaches allow recovery of sample phase enabling quantitative label-free imaging of live cells [250]. Lens-free computational imaging has been applied for sizing of ambient PM [251]. Another computational imaging technique, light field microscopy (LFM) [252], enables capture of three-dimensional information in a single camera exposure, allowing both high-speed particle tracking [253] and real-time three-dimensional visualization of biological systems [254]. Although light field methods necessitate a reduction in spatial resolution, recent work has combined LFM with single-molecule fluorescence techniques achieving an isotropic localization precision of approximately 20 nm over the volume of an entire eukaryotic cell at many frames per second [255]. Combined with suitable fluorescent labelling techniques, these approaches may allow real high-speed time-lapse imaging of intracellular uptake of aerosols. New and emerging SRM techniques continue to push the spatial resolution possible using fluorescence techniques closer to that of EM. The MINFLUX [256] method can achieve nanometre-level resolution by localizing individual fluorophores. This level of resolution has the potential to further reveal new structural characteristics of cells such as the structure of protein complexes.

Aside from the development of new techniques, further improvements to hardware can be expected to improve existing imaging methods. Recent years have seen significant improvements in digital camera technology with the development of scientific complementary metal oxide semiconductor (sCMOS) cameras with higher resolution sensors and higher frame rates than traditional CCD-based systems. sCMOS cameras are now the preferred option for most camera-based FM systems from super-resolution techniques such as SMLM [257] and SIM [203] to LSM [258]. The most recent generation of sCMOS cameras have an increased quantum efficiency allowing for improved imaging at low light levels raising the possibility of reducing sample excitation to limit imaging artefacts associated with photobleaching and phototoxicity [259]. Similarly, the development of new, brighter, more photostable fluorescent dyes [260] promises to improve image quality and facilitate less invasive imaging.

Correlative microscopy techniques [261] combine different imaging modalities to provide a more complete picture of structural and functional characteristics. Most commonly, EM and FM are applied together to combine high-resolution ultrastructure with the biomolecular specificity of fluorescent labelling. Such an approach has been applied to visualize macrophages within an *ex vivo* mouse lung section [262]. Through the development of new sample processing, imaging and analysis workflows, correlative imaging using the diverse array of chemical, structural and functional bioimaging techniques offers exciting possibilities for *in vitro* toxicology. In parallel with the development of image acquisition hardware, recent years have seen the emergence of new computational methods with the potential to analyse biological images. Notably, deep learning [263] methods are particularly well suited to tasks such as object segmentation and binary classification. These approaches, along with more traditional computer vision methods, offer huge potential to develop more effective image-based toxicological screening assays [264] and detect patterns in image data to inform our understanding of toxicological mechanisms.

## 5. Conclusion and outlook

The models and exposure systems described in this article enable laboratory-based respiratory toxicology studies which more closely mimic the exposure of cells to sources of PM under real-world conditions. Visualizing and analysing the stages of the cell–particle interaction processes within these large, complex models requires effective application of a range of biological imaging techniques and, critically, matching the properties of the image system to the characteristics of the model system. By considering the properties of different *in vitro* respiratory models along with the ability of imaging techniques to probe them, we aim to provide a basis for the development of more effective image-based toxicological studies and a stimulus to scientists and engineers developing new models and new image-based analytical methods.

The precise mechanisms involved in respiratory toxicology are complicated and often unclear at present. Further investigations are needed to elucidate the disease-specific cell and tissue mechanisms by which inhalation of airborne particles leads to adverse impacts on human health. One of the most exciting fields of study in respiratory toxicology lies in investigating the integrative role of cell biomechanics, signal transduction, induction of oxidative stress and inflammation and the long-term impacts on gene regulation, senescence and tissue remodelling in the onset of respiratory pathologies. Interrogation of relevant *in vitro* respiratory models exposed to well-characterized PM using high-resolution imaging techniques, combined with data from other toxicological screens and omics analysis, can provide important new insights into the underlying molecular and cellular processes. Such empirical laboratory-based studies offer a means by which to gather evidence to underpin the development of effective targeted air quality legislation and to move beyond simple mass-based metrics to a more nuanced consideration of PM component and sources. This knowledge is essential for designing mitigation measures that target the most hazardous components of air pollution and to improve public health globally.

## Data Availability

This article has no additional data.
